# Placentae for Low Birth Weight Piglets Are Vulnerable to Oxidative Stress, Mitochondrial Dysfunction, and Impaired Angiogenesis

**DOI:** 10.1155/2020/8715412

**Published:** 2020-05-25

**Authors:** Chengjun Hu, Yunyu Yang, Ming Deng, Linfang Yang, Gang Shu, Qingyan Jiang, Shuo Zhang, Xiaozhen Li, Yulong Yin, Chengquan Tan, Guoyao Wu

**Affiliations:** ^1^Guangdong Provincial Key Laboratory of Animal Nutrition Control, National Engineering Research Center for Breeding Swine Industry, Institute of Subtropical Animal Nutrition and Feed, College of Animal Science, South China Agricultural University, Guangzhou, Guangdong 510642, China; ^2^Guangdong Yihao Foodstuffs Co., Ltd., Guangzhou, Guangdong 510642, China; ^3^Yunnan Yin Yulong Academician Workstation, Yunnan Xinan Tianyou Animal Husbandry Technology Co., Ltd., Kunming, Yunnan 650032, China; ^4^National Engineering Laboratory for Pollution Control and Waste Utilization in Livestock and Poultry Production, Institute of Subtropical Agriculture, Chinese Academy of Sciences, Changsha, Hunan 410125, China; ^5^Department of Animal Science, Texas A&M University, College Station, Texas 77843-2471, USA

## Abstract

Intrauterine growth restriction (IUGR) is associated with fetal mortality and morbidity. One of the most common causes of IUGR is placental insufficiency, including placental vascular defects, and mitochondrial dysfunction. In addition, a high level of oxidative stress induces placental vascular lesions. Here, we evaluated the oxidative stress status, mitochondrial function, angiogenesis, and nutrient transporters in placentae of piglets with different birth weights: <500 g (L), 500–600 g (LM), 600–700 g (M), and >700 g (H). Results showed that placentae from the L group had higher oxidative damage, lower adenosine triphosphate and citrate synthase levels, and lower vascular density, compared to those from the other groups. Protein expression of angiogenic markers, including vascular endothelial cadherin, vascular endothelial growth factor A, and platelet endothelial cell adhesion molecule-1, was the lowest in the L group placentae compared to the other groups. In addition, the protein levels of glucose transporters GLUT1 and GLUT3 were downregulated in the L group, compared to the other groups. Furthermore, oxidative stress induced by H_2_O_2_ inhibited tube formation and migration in porcine vascular endothelial cells. Collectively, placentae for lower birth weight neonates are vulnerable to oxidative damage, mitochondrial dysfunction, and impaired angiogenesis.

## 1. Introduction

Intrauterine growth restriction (IUGR) is a pathological complication with reduced fetal growing during pregnancy. IUGR is diagnosed when fetal weight is below the 10th percentile for gestational age [1], and low birth weight in humans is defined by the World Health Organization as the birth weight less than 2.5 kg [2]. This complication was associated with fetus and newborn mortality, abnormal neurodevelopment, and morbidity in humans [3, 4]. Animals (such as piglets) with a low birth weight had higher rates of morbidity and mortality before weaning [5] and a slower rate of growth after weaning [6]. Reduction in the flow of blood from the placenta to the fetus [7] and maternal nutritional intervention, such as limited or excess dietary protein levels [8, 9] and low dietary energy levels [10], could increase the birth of IUGR piglets. Although studies have provided evidence for the comprehension of molecular bases for IUGR, the mechanisms underlying the occurrence of IUGR remain largely unknown. Elucidating this mechanism is important for formulating nutritional strategies that can promote fetal growth during pregnancy [11].

One of the main causes of IUGR is the placental insufficiency in distributing enough nutrients and oxygen to the fetus. Increased oxidative stress level was observed in the placenta from low birth weight mammals [12]. For instance, a decrease in glutathione (GSH) concentration [13] and an increase in oxidative DNA damage occur in IUGR placentae of humans [14]. Placental reactive oxygen species (ROS) are derived from a variety of sources, such as mitochondrial respiratory chain, endoplasmic reticulum dysfunction, and enzymes (xanthine oxidase, endothelial nitric oxide synthase, and NADPH oxidase) [15]. However, the underlying mechanisms regarding increased oxidative stress levels in the placenta from low birth weight mammals are largely unknown. Placental blood vessels are important for fetal growth and development [16]. Placentae with high vascular density can help to increase maternal-fetal nutrients, respiratory gases, and waste exchanges, thus promoting fetal growth and survival [17]. Vascular development is regulated by vascular endothelial growth factor A (VEGF-A), and the level of this growth factor and the placental vascular density are decreased in low birth weight fetus placentae [17, 18]. Previous studies showed that oxidative stress can cause vascular dysfunction in the placenta [19, 20], suggesting that oxidative stress in the placenta may be involved in the development of IUGR offspring through modulating placental vessel development. Citrate synthase regulates adenosine triphosphate (ATP) generation in mitochondria via catalyzing the first step of the tricarboxylic acid cycle [21]. Evidence shows that reduced citrate synthase activity is associated with mitochondrial dysfunction [22]. Mitochondria also play an important role in trophoblast proliferation, invasiveness, and placental insufficiency, suggesting that placental mitochondrial dysfunction may be responsible for IUGR [23, 24].

Pigs have been selected as a model for human disease and clinical medicine investigations due to their physiological characteristics which are similar to humans [25, 26]. Therefore, the aims of this study were to determine ROS levels, mitochondrial function, and angiogenesis in placentae of piglets with different birth weights.

## 2. Materials and Methods

The experimental design and procedure presented in this study were reviewed and approved by the Animal Care and Use Committee of the Institute of Subtropical Agriculture, Chinese Academy of Sciences, under ethic approval number ISA-2018-045.

### 2.1. Animals and Study Design

The gilts (Guangdong small-ear spotted pig) used in this study were obtained from the farm of Guangdong Yihao Foodstuffs Co., Ltd., Guangdong province, China. Gilts were fed 1.2-2.5 kg of a common corn and soybean meal-based gestation diet (Supplemental Table 1). During gestation, gilts were housed individually in conventional stalls (2.0 × 0.7 × 1.0 m). Gilts were fed twice daily at 07.00 and 16.00 h and had *ad libitum* access to water through nipple drinkers. Gilts were cleaned with warm water and moved to the farrowing rooms on day 110 of gestation and housed individually in fully slatted farrowing crates (2.2 m × 1.5 m). Their backfat thickness was measured at the P2 position at day 110 of gestation using A-mode ultrasonography (Renco Lean-Meater®, Minneapolis, MN, USA.). Body weight was recorded at day 110 of gestation.

### 2.2. Data Collection and Sampling

In order to match individual piglets with their placentae, gilts were observed when farrowing. When piglets were born, the umbilical cord next to the piglet was immediately tied with a silk line labeled with a numbered tag (to indicate the birth order of the piglet), then the umbilical cord was cut and allowed to retract into the birth canal [27]. The birth weight and numerical order of newborn piglets were recorded immediately. The placentae were weighed after they were expelled. Then, approximately 5 g of each placenta (3 to 4 cm from the cord insertion point) was immediately collected and snap-frozen in liquid nitrogen [28]. Another fresh placental tissue was immediately fixed in 4% paraformaldehyde. Placental efficiency was calculated by dividing piglet weight by placental weight. In this study, the mean birth weight of the 178 piglets was 654 ± 11 g (means ± standard error). The placentae were assigned to four groups according to piglet birth weight: <500 g (lower birth weight, L), 500–600 g (lower-medium birth weight, LM), 600–700 g (medium birth weight, M), and >700 g (higher birth weight, H).

### 2.3. Oxidative Stress Parameters

Placental ROS, malondialdehyde (MDA), superoxide dismutase (SOD), GSH, and 8-hydroxy-2′-deoxyguanosine (8-OHdG) were determined using their commercial kits (Nanjing Jiancheng Bioengineering Institute, Nanjing, China).

Placental total protein concentrations were measured according to the instructions of the bicinchoninic acid (BCA) protein assay kit (Beyotime, Beijing, China). The placental ROS, MDA, SOD, GSH, and 8-OHdG levels were normalized to the placental total protein.

### 2.4. ATP, NAD^+^, and NADH Levels in Placentae

Placental ATP, nicotinamide adenine dinucleotide (NAD^+^), nicotinamide adenine dinucleotide, and reduced form (NADH) levels were determined using commercial kits (Beyotime, Beijing, China) according to the manufacturer's instructions. Placental ATP, NAD^+^, and NADH levels were normalized to the placental total protein.

### 2.5. Citrate Synthase, Complex I, and Complex III Activities

Citrate synthase activity in the placenta was determined using a commercial kit (Nanjing Jiancheng Bioengineering Institute, Nanjing, China) according to the manufacturer's instructions. NADH ubiquinone oxidoreductase (complex I) and ubiquinol cytochrome reductase (complex III) activities were assessed using commercial kits (Cominbio Co., Suzhou, China) according to the manufacturer's instructions. Placental citrate synthase, complex I, and complex III activities were normalized to the placental total protein.

### 2.6. Mitochondrial DNA (mtDNA) Copy Number

Total genomic DNA was extracted from the placenta using the QIAamp DNA Mini Kit (QIAGEN, USA). Primers for mitochondrial cytochrome b (Cytb) and 18S ribosomal RNA (18S rRNA) genes were used for determination of mtDNA content [29] and are listed in supplemental Table 2.

### 2.7. Placental Vascular Density

Placental tissues fixed in 4% paraformaldehyde were paraffin-embedded and sectioned at 5 *μ*m thickness [30], followed by staining with hematoxylin and eosin (H&E). The placental vessels in these areas were also traced using a projecting microscope (Olympus CX41, Japan). Placental vascular areas were then quantified as previously described [31].

### 2.8. RNA Extraction, Complementary DNA Synthesis, and Real-Time Quantitative RT-PCR

Total RNA was extracted from the placental tissue using the TRIzol reagent (Invitrogen, Carlsbad, CA, USA). The concentration of RNA was quantified using a NanoDrop® ND-1000 (Thermo Fisher, Wilmington, DE, USA). The RNA integrity was determined using 1% agarose gel electrophoresis, which showed 5S, 18S, and 28S rRNA bands. Complementary DNA (cDNA) was synthesized using the PrimeScript RT reagent kit (Takara, Dalian, China) according to the manufacturer's instructions. Primers were designed using Primer 3 and are listed in Supplemental Table 2. The mRNA levels of target genes were determined as previously described [32]. The 18S rRNA was used as the housekeeping gene to normalize the mRNA levels of the target genes.

### 2.9. Western Blotting

Total proteins were extracted from placental tissues using the protein extraction kit (Beyotime, Beijing, China) according to the manufacturer's guide and then separated by SDS-PAGE and blotted onto PVDF membranes. Blots were then incubated overnight at 4°C with each of the following primary antibodies: VEGF-A polyclonal antibody (19003-1-AP; Proteintech, USA, 1 : 1000 dilution), vascular endothelial-cadherin (VE-cadherin) polyclonal antibody (2500T; CST, USA, 1 : 1000 dilution), glucose transporter 1 (GLUT1) antibody (bs-0472R; Bioss, China, 1 : 1000 dilution), GLUT3 antibody (bs-1207R; Bioss, China, 1 : 1000 dilution), sodium-coupled neutral amino acid transporter 2 (SNAT2) antibody (bs-12125R; Bioss, China, 1 : 1000 dilution), and *β*-actin (4970; CST, USA, 1 : 1000 dilution). The density of bands was quantified using the ImageJ software (National Institutes of Health, Bethesda, MD) and then normalized to *β*-actin content.

### 2.10. Immunofluorescence

Placental tissues fixed in 4% paraformaldehyde were paraffin-embedded and sectioned at 5 *μ*m thickness for platelet endothelial cell adhesion molecule-1 (CD31) immunofluorescence. The method was performed as described previously [33].

### 2.11. Cell Culture

Porcine vascular endothelial cells (PVECs) were obtained from the Cell Bank of the Chinese Academy of Sciences (Shanghai, China). PVECs grew in 1640 medium with 10% fetal bovine serum, 100 U/mL penicillin, and 100 *μ*g/mL streptomycin at 37°C in 5% CO_2_ atmosphere.

### 2.12. Tube Formation and Wound Healing Assay In Vitro

PVECs were seeded in 96-well plates precoated with 50 *μ*L Matrigel (BD company, USA) at a density of 4 × 10^4^ cells per well. Images were captured 6 h after treatment using an Olympus inverted microscope (40x) and analyzed using ImageJ software. PVEC monolayers were wounded with a 10 *μ*L pipette tip and maintained for 24 h in basal medium with or without 200 *μ*M H_2_O_2_. Wounded areas were captured using an Olympus inverted microscope and quantified using the ImageJ software.

### 2.13. Enzyme-Linked Immunosorbent Assay

The culture media were collected, and VEGF-A secretion was measured through ELISA (CSB-E12053p, Cusabio, Wuhan, China, https://www.cusabio.com/) according to the manufacturer′s protocol.

### 2.14. Statistical Analysis

Data are presented as mean ± SEM and were statistically analyzed using one-way ANOVA and Duncan's multiple-range test in SPSS 20.0 (SPPS Inc., Chicago, IL). Tamhane's T2 test was used to assess variance heterogeneity. Pearson's correlation coefficient was used to analyze the correlation between piglet birth weight and placental vascular density. *P* values < 0.05 were taken to indicate statistical significance.

## 3. Results

### 3.1. Selected Characteristics of Gilts and Piglets

The placentae were allocated to four groups according to piglet birth weight (Table 1). As expected from the study design, a difference was observed in the birth weight of piglets among the four groups (*P* < 0.01). The placental weight increased as the piglet birth weight increased (*P* < 0.01). Placental efficiency, maternal backfat thickness, and maternal body weight at day 110 of gestation did not differ among the four groups.

Metabolic and mechanistic data, described below, were obtained in 10, 10, 9, and 9 placentae from the L, LM, M and H groups, respectively.

### 3.2. Oxidative Stress Levels in Placentae

**Figure 1:**
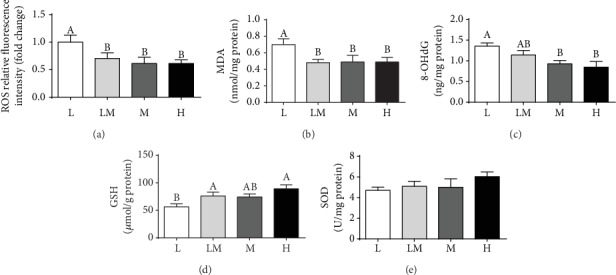
Placental oxidative stress levels. ROS (a), MDA (b), 8-OHdG (c), GSH (d), and SOD (e) levels were normalized to the placental total protein. Data are presented as mean ± SEM, and the numbers of replicates in the L, LM, M, and H groups were 10, 10, 9, and 9, respectively. Different letters indicate significant differences at *P* < 0.05. L: <500 g group; LM: 500-600 g group; M: 600-700 g group; H: >700 g group.

As shown in Figure 1, placental ROS, MDA, and 8-OHdG levels were higher in the L group than in the other groups (*P* < 0.05). A decrease in the GSH level was observed in the L group, compared to the LM or the H group (*P* < 0.05). However, no differences were observed in placental ROS, MDA, 8-OHdG, GSH, and SOD levels among the LM, M, and H groups.

### 3.3. ATP Levels and Mitochondrial Function in Placentae

**Figure 2:**
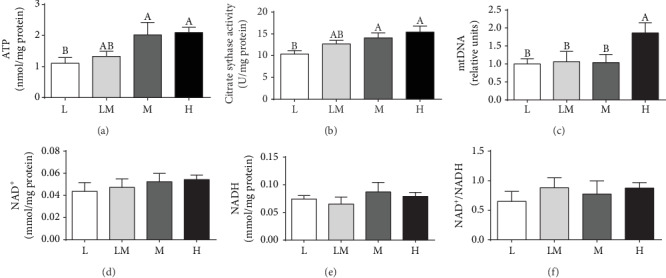
Mitochondrial function in the placentae. Mitochondrial function was estimated by ATP levels (a), citrate synthase activity (b), mitochondrial DNA (mtDNA) copy number (c), NAD^+^ (d), NADH (e), and the ratio of NAD^+^/NADH (f). Data are presented as mean ± SEM, and the numbers of replicates in the L, LM, M, and H groups were 10, 10, 9, and 9, respectively. Different letters indicate significant differences at *P* < 0.05.

Placental ATP levels (Figure 2(a)) and citrate synthase activity (Figure 2(b)) were lower in the L group than in the M or the H group (*P* < 0.05). The highest amount of mtDNA (Figure 2(c)) was observed in the H group (*P* < 0.05). No differences were observed in the NAD^+^/NADH ratio, as well as the NAD^+^ and NADH levels among the four groups of piglets.

### 3.4. Activities of Mitochondrial Complex I and Complex III in Placentae

**Figure 3:**
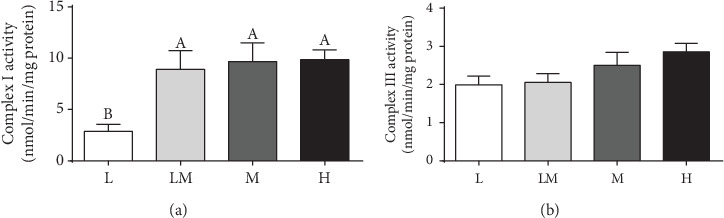
Activities of placental mitochondrial complex I (a) and complex III (b). Data are presented as mean ± SEM, and the numbers of replicates in the L, LM, M, and H groups were 10, 10, 9, and 9, respectively. Different letters indicate significant differences at *P* < 0.05.

Complex I activity in the L group was the lowest (*P* < 0.05), compared with that in the other groups of piglets (Figure 3(a)). No difference was observed in complex III activity among the four groups of piglets (Figure 3(b)).

### 3.5. Placental Vessel Density

**Figure 4:**
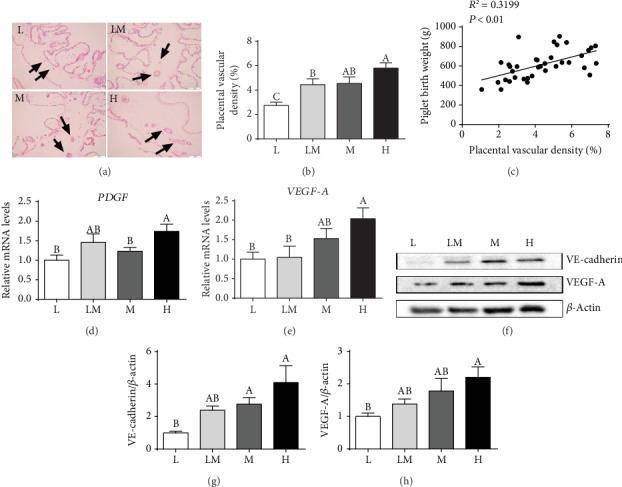
The vessel density distribution in the placentae. The hematoxylin & eosin method (a) was used to examine blood vessel density in placental tissues, and the black arrows indicate placental blood vessels (bar = 100 *μ*m). (b) The percentage of blood vessels in the placental tissues. The numbers of replicates in the L, LM, M, and H groups were 10, 10, 9, and 9, respectively. Correlation between placental blood vessel density and piglet birth weight (c). mRNA levels of *PDGF* (d) and *VEGF-A* (e); *n* = 8 per group. Western blotting (f) and protein expression levels of VE-cadherin (g) and VEGF-A (h). Data are presented as mean ± SEM, and the numbers of replicates in the L, LM, M, and H groups were 10, 10, 9, and 9, respectively. Different letters indicate significant differences at *P* < 0.05.

As shown in Figure 4, H&E staining demonstrated the vessel distributions in placental tissues (Figure 4(a)). Placental vessel density is presented in Figure 4(b), showing a decrease (*P* < 0.05) in the vasculature in the L group compared to other groups. In addition, a positive correlation (*P* < 0.05) was observed between the placental vascular density and piglet birth weight (Figure 4(c)). The mRNA levels of platelet-derived growth factor C (*PDGF-C*) (Figure 4(d)) and *VEGF-A* (Figure 4(e)) were higher (*P* < 0.05) in the H group than in the L group. The protein abundance of VE-cadherin and VEGF-A (Figures 4(f)–4(h)) was decreased (*P* < 0.05) in the L group, compared to the H group.

**Figure 5:**
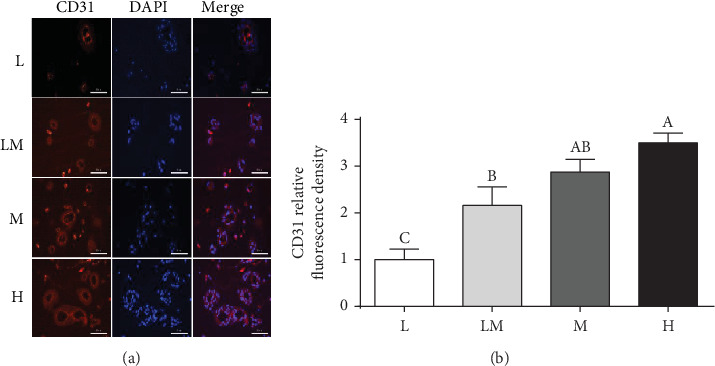
CD31 immunofluorescence staining (a) in placentae. (b) Summarized data. Data are presented as mean ± SEM, and the numbers of replicates in the L, LM, M, and H groups were 10, 10, 9, and 9, respectively. Different letters indicate significant differences at *P* < 0.05. Bar = 50 *μ*m.

Moreover, placentae in the L group showed a decrease (*P* < 0.05) in the immunostaining intensity of CD31, compared to the other groups of piglets (Figures 5(a) and 5(b)).

### 3.6. The mRNA and Protein Abundance of Placental Transporters

**Figure 6:**
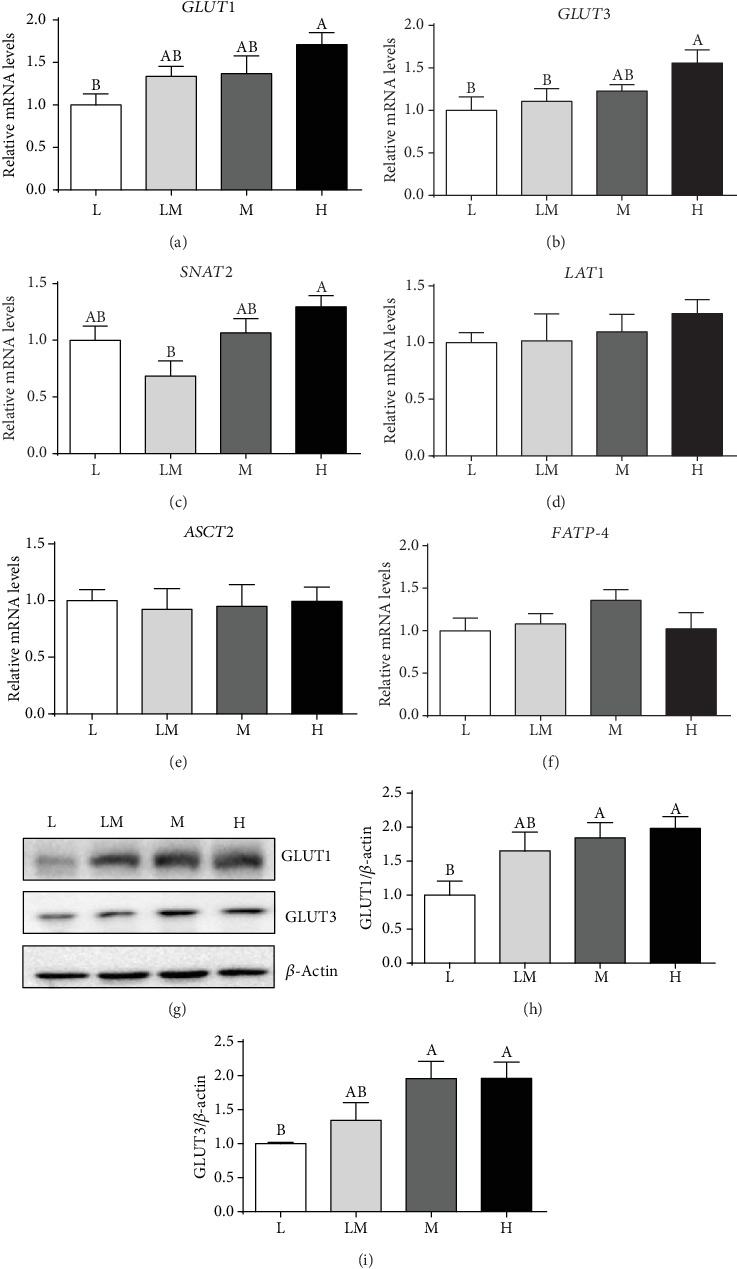
The expression levels of transporters in the placentae. The mRNA levels of *GLUT1*, *GLUT3*, *SNAT2*, *LAT1*, *ASCT2*, and *FATP-4* (a–f) were normalized using 18S as an internal control. Western blotting (g) and protein expression levels of GLUT1 (h) and GLUT3 (i). Different letters indicate significant differences at *P* < 0.05. Data are presented as mean ± SEM, and the numbers of replicates in the L, LM, M, and H groups were 10, 10, 9, and 9, respectively.

As shown in Figure 6, the L group showed a decrease (*P* < 0.05) in the mRNA and the protein abundance of GLUT1 and GLUT3, compared to the H group. A higher (*P* < 0.05) mRNA level of *SNAT2* was observed in the H group than in the LM group. However, no differences (*P* > 0.05) were observed in the mRNA levels of the L-type (large neutral) amino acid transporter 1 (*LAT1*), ASC amino acid transporter 2 (*ASCT2*), and fatty acid transport protein 4 (*FATP-4*) among the four groups of piglets.

### 3.7. Oxidative Stress Induced by H_2_O_2_ Inhibited Tube Formation and Migration In Vitro

**Figure 7:**
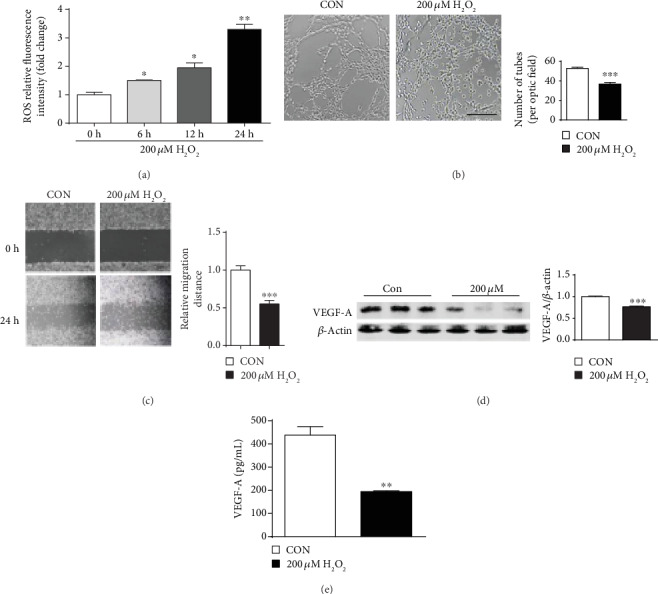
Oxidative stress induced by H_2_O_2_ inhibited tube formation and migration in porcine vascular endothelial cells (PVECs). (a) ROS generation in PVECs treated with 200 *μ*M H_2_O_2_ for 6, 12, and 24 h. *n* = 3. (b) Representative images of tube formation by PVEC cells, treated with 200 *μ*M H_2_O_2_ for 24 h (right) or in standard medium (left). Cells were seeded at a density of 4 × 10^4^ cells/well on a plate precoated with Matrigel and cultured for 6 h. *n* = 5. (c) Representative micrographs of wound healing experiments. PVEC cells were maintained for 24 h in basal medium (left) or treated with 200 *μ*M H_2_O_2_ (right). *n* = 3. (d) VEGF-A protein levels were detected by Western blotting in cells after treatment with 200 *μ*M H_2_O_2_ for 24 h. VEGF-A protein levels were detected by Western blotting after cells under treatment with 200 *μ*M H_2_O_2_ for 24 h. *n* = 3. (e) VEGF-A levels were measured by ELISA in the culture medium of cells under treatment with 200 *μ*M H_2_O_2_ for 24 h. *n* = 5. Data are presented as mean ± SEM. The differences between the two groups were analyzed by Student's *t*-test. All experiments were performed in triplicate. ^∗^*P* < 0.05; ^∗∗^*P* < 0.01; ^∗∗∗^*P* < 0.001.

As shown in Figure 7, ROS levels increased (*P* < 0.05) in PVECs after treatment with 200 *μ*M H_2_O_2_. In addition, oxidative stress induced by 200 *μ*M H_2_O_2_ reduced (*P* < 0.05) PVEC tube formation and migration. Treatment with 200 *μ*M H_2_O_2_ also decreased (*P* < 0.05) the protein levels of VEGF-A in PVECs and in cell culture media.

## 4. Discussion

Placenta plays an important role in fetal growth. The present study demonstrated increased oxidative damage, decreased mitochondrial function, impaired angiogenesis, and downregulated protein levels of glucose transporters in placentae for low birth weight piglets. Furthermore, compared to the L group, placental ATP level, citrate synthase activity, mtDNA content, mitochondrial complex I activities, and vascular density were higher, whereas ROS, MDA, and 8-OHdG levels were lower in the H group. These findings indicated that placentae for low birth weight neonates were vulnerable to oxidative stress, mitochondrial dysfunction, and impaired angiogenesis, which might contribute to the occurrence of low birth weight piglets during pregnancy.

Oxidative stress is an important factor for many complications during pregnancy. Physiological levels of ROS are important for placental development and fetal growth, whereas overproduction can result in pregnancy complications including IUGR [34]. In the present study, we found excessive ROS production and lower GSH level in placentae for low birth weight piglets, which was in line with results by Luo et al. [12]. Excessive ROS accumulation causes oxidative damage to lipids and DNA, primarily in the form of lipid peroxidation and DNA oxidation [14]. The higher MDA and 8-OHdG levels in lower birth weight placentae further confirmed greater oxidative damage in these compromised placentae. Pigs can be regarded as a good animal model for better understanding of the IUGR syndrome in humans [25]. Consistent with our results, human studies also showed that antioxidant activities decreased and oxidative damage increased in IUGR placentae [13, 14]. Mitochondria are not only the sites for ATP and ROS generation [35] but also a target of ROS attack [36]. Therefore, the mitochondrial function in placentae was evaluated. Our results showed that placental mitochondrial function was reduced in the L group, as evidenced by decreased ATP and citrate synthase levels, which was consistent with the results of published work [12]. The mtDNA content in placenta is recognized as a measure of the mitochondrial content and a novel biomarker of systemic mitochondrial dysfunction [37]. Evidence showed a higher mtDNA content in human IUGR placentae [38, 39]. In contrast, Luo et al. reported that the mtDNA content was decreased in swine IUGR placentae [12]. Our results demonstrated that the mtDNA content was highest in the H group placentae, which was consistent with Diaz et al. (2014), who reported that placentae of small gestational-age infants had a lower mtDNA content [40]. Collectively, these findings suggest that placental mitochondrial function was decreased in the lower birth weight placentae. Mitochondrial complex I and complex III are the major sites for ROS generation, and most of free radical oxygen species are produced by complex I [41]. To define factors that affect ROS production in the lower birth weight placentae, the activities of subunits encoding the complexes of the electron transport chain were measured. Our results showed that the activity of mitochondrial complex I was decreased in the L group compared to the other groups, suggesting that a decrease in the activities of mitochondrial complex I might contribute to increased ROS levels in the low birth weight placentae.

Placental blood vessels are responsible for the exchanges of gases, nutrients, and wastes between the mother and its fetus, which is essential for fetal growth. Poor vascularization of the placenta leads to IUGR and even fetal death [42, 43]. Therefore, the placental vessel density was determined in the present study. We found that among our study piglets, placental vessel density was the lowest in the L group and was the highest in the H group than in the other groups, which was in line with Song et al. (2018), who reported that the density of the placental vessels was lower in low birth weight piglets [17]. CD31 is a biomarker of endothelial cells in blood vessels [44]. The decreased CD31 immunofluorescence staining further demonstrated that the placental vessel density in the L group was the lowest among all the four groups of piglets. Moreover, we also observed that piglet birth weight was positively associated with placental vascular density. The VE-cadherin is a junction adhesion molecule uniquely expressed in endothelial cells and exerts an important role in maintaining vascular integrity [45], and VEGF-A is a major driver of blood vessel formation. Here, we found that the protein abundance of VE-cadherin and VEGF-A was the lowest in the L group placentae among the four groups of piglets, suggesting that decreased VE-cadherin and VEGF-A protein expression in placentae may result in reduced vessel density in low birth weight placentae. In line with our study, Ravikumar et al. (2016) also reported that the placental *VEGF* mRNA level was lower in IUGR placentae compared to the appropriate gestational age in humans [46]. Interestingly, oxidative stress induced by H_2_O_2_ inhibited PVEC tube formation and migration in vitro, as well as VEGF-A secretion. Collectively, these results suggest that increased oxidative stress levels might contributed to reduced angiogenesis in placentae.

Glucose and amino acids are substrates for both fetal growth and placental development [7]. Therefore, transporters related to the transfer of glucose and amino acids from mother to fetus were determined. Placental GLUT1 and GLUT3 are responsible for glucose transport, and SNAT2 facilitates the uptake of small amino acids including alanine, glycine, and serine [47]. Previous studies showed that decreased placental glucose and amino acid transporters were associated with IUGR [48, 49] and that GLUT1 expression in the syncytial microvilli of human IUGR placentae was significantly reduced [50]. Similarly, we found that the mRNA and the protein abundance of GLUT1 and GLUT3 was decreased in the L group placentae, demonstrating that a reduction in placental glucose transport was associated with IUGR. The mRNA level of *SNAT2* was lower in the LM group placenta than in the H group placenta, which is consistent with results of a previous study in humans [51]. An increased oxidative stress level results in reduced glucose uptake and reduced GLUT1 expression in human placentae [52]. This will compromise the availability of glucose and amino acids in the fetus for metabolic utilization, including intracellular protein synthesis and antioxidative reactions [53]. However, further studies are needed to investigate the effect of the oxidative stress level on placental glucose transport in pigs.

## 5. Conclusion

Our results demonstrated increased oxidative damage, decreased mitochondrial function, impaired angiogenesis, and downregulated glucose transporters in placentae for low birth weight piglets. In addition, low birth weight piglet placentae had lower protein abundance of VE-cadherin and VEGF-A. Decreased angiogenesis in the placentae may be attributed to the increased oxidative stress level. Our findings provide new insight into the role of placental function in fetal growth and suggest that modulation of oxidative stress and angiogenesis in placentae might provide a novel therapeutic approach to improving fetal growth in mammals.

## Figures and Tables

**Table 1:** Selected characteristics of gilts and piglets^1^.

Items	L	LM	M	H	SEM	*P* value
Number of gilts (*n*)	20	21	41	23		
Number of placentae (*n*)	25	41	50	62		
Piglet birth weight (g)	442.4^d^	555.9^c^	646.5^b^	810.5^a^	10.6	<0.01
Placental weight (g)	107.1^c^	124.4^b^	134.4^b^	158.3^a^	2.9	<0.01
Placental efficiency^2^	4.6	4.8	5.1	5.4	0.1	0.07
Backfat thickness (mm)^3^	33.2	31.1	32.7	32.8	0.3	0.20
Body weight (kg)^4^	108.0	107.3	106.0	108.7	0.8	0.59

^1^L, LM, M, and H indicate piglet birth weight < 500 g, 500 g-600 g, 600-700 g, and >700 g, respectively. ^2^Placenta efficiency = piglet weight (g)/placenta weight (g). ^3,4^Measured at day 110 of gestation. ^a–d^Values are means with pooled SEM. Within a row, means not sharing the same superscript letters differ significantly (*P* < 0.05).

## Data Availability

The data used to support the findings of this study are available from the corresponding author upon request.

## Supplementary Materials

Supplementary Materials Supplementary Table 1: composition and nutrient levels of the experimental diets. Supplemental Table 2: primers used for real-time PCR. ASCT2: ASC amino acid transporter 2; FATP-4: fatty acid transport protein 4; GLUT1/3: glucose transporters 1 and 3; PDGF-C: platelet-derived growth factors C; LAT1: L-type (large neutral) amino acid transporter 1; SNAT2: sodium-dependent neutral amino acid transporter 2; VEGF-A: vascular endothelial growth factor A; MT-CYB: mitochondrially encoded cytochrome b; 18S rRNA: 18S ribosomal RNA.
