# Supplementary material for: Placentae for Low Birth Weight Piglets Are Vulnerable to Oxidative Stress, Mitochondrial Dysfunction, and Impaired Angiogenesis
Source: Oxid Med Cell Longev. 2020 May 25;2020:8715412. doi: 10.1155/2020/8715412 (PMC7267862; doi:10.1155/2020/8715412)
Supplement: Supplementary Materials — Supplementary Table 1: composition and nutrient levels of the experimental diets. Supplemental Table 2: primers used for real-time PCR. ASCT2: ASC amino acid transporter 2; FATP-4: fatty acid transport protein 4; GLUT1/3: glucose transporters 1 and 3; PDGF-C: platelet-derived growth factors C; LAT1: L-type (large neutral) amino acid transporter 1; SNAT2: sodium-dependent neutral amino acid transporter 2; VEGF-A: vascular endothelial growth factor A; MT-CYB: mitochondrially encoded cytochrome b; 18S rRNA: 18S ribosomal RNA. [file 8715412.f1.docx]

**Supplemental Table 1.** Composition and nutrient levels of the experimental

diets (as-fed basis)

| Ingredient, % | d 1 to d 85 | d 85 to delivery |
| --- | --- | --- |
| Corn | 43.09 | 48.69 |
| Soybean meal | 7.80 | 19.00 |
| Wheat bran | 20.00 | 17.00 |
| Barley | 10.00 | - |
| Rice bran meal | - | 10.00 |
| Soybean hull | 15.00 | - |
| Mountain flour | - | 0.4 |
| CaHCO_3_ | - | 0.8 |
| Premix^1^ | 4.11 | 4.11 |
| Total | 100.00 | 100.00 |
| Chemical composition^2^ |  |  |
| Digestible energy, MJ/kg | 11.83 | 12.65 |
| CP, % | 12.64 | 16.70 |
| CF, % | 8.51 | 4.10 |
| EE, % | 2.95 | 4.12 |
| Ca, % | 1.00 | 1.21 |
| Lys, % | 0.56 | 0.79 |
| Met+Cys  Trp  Thr | 0.52  0.14  0.48 | 0.61  0.20  0.63 |

^1^Premix provided for 1 kg of complete diet: Cu,75 mg; Fe,750 mg; Se, 0.20 mg; Zn, 750 mg; Mn, 187 mg; vitamin A, 150,000 IU; vitamin B_2_, 140 mg; vitamin D_3_, 25,000 IU; vitamin E, 72 IU; vitamin K_3_, 35 mg; vitamin B_2_, 140 mg; vitamin B_6,_ 70mg; calcium pantothenate, 350 mg; niacin, 500 mg; and vitamin B_12_, 0.4 mg.

^2^ Calculated chemical concentrations using values for feed ingredients from National Research Council (2012).

**Supplemental Table 2.** Primers used for real-time PCR

| Genes | Primers | Primers Sequences (5^，^to 3^，^) | Size (bp) |
| --- | --- | --- | --- |
| *ASCT2* | Forward | GATTGTGGAGATGGAGGATGTGG | 128 |
|  | Reverse | TGCGAGTGAAGAGGAAGTAGATGA |  |
| *LAT1* | Forward | TTTGTTATGCGGAACTGG | 155 |
|  | Reverse | AAAGGTGATGGCAATGAC |  |
| *SNAT2* | Forward | TACTTGGTTCTGCTGGTGTCC | 212 |
|  | Reverse | GTTGTGGGCTGTGTAAAGGTG |  |
| *FABP-4* | Forward | TGGAAACTTGTCTCCAGTG | 147 |
|  | Reverse | GGTACTTTCTGATCTAATGGTG |  |
| *PDGF-C* | Forward | GGAGTACAAGATCCCCAGCA | 173 |
|  | Reverse | TCCAGCCCAAATCTCTCATC |  |
| *VEGF-A* | Forward | CCTCGGAGCGGAGAAAGCAT | 126 |
|  | Reverse | TGTCACATCTGCAAGTACGTTCG |  |
| *GLUT1* | Forward | CCTTCAGCCAGCAGTGATG | 179 |
|  | Reverse | AGCGTGGGATGTGGGTAAAG |  |
| *GLUT3* | Forward | GCCTTGACCTTTCCCATAGACA | 111 |
|  | Reverse | CTACTTCCACCCAGAGCAAAGT |  |
| *MT-Cytb* | Forward | ATGAAACATTGGAGTAGTCCTACTATTTACC | 149 |
|  | Reverse | CTACGAGGTCTGTTCCGATATAAGG |  |
| *18S rRNA* | Forward | GGTAGTGACGAAAAATAACAATACAGGAC | 141 |
|  | Reverse | ATACGCTATTGGAGCTGGAATTACC |  |
